# An Acceptance and Commitment Therapy Smartphone Application for Erectile Dysfunction: A Feasibility Study

**DOI:** 10.1016/j.curtheres.2023.100728

**Published:** 2023-11-17

**Authors:** Junichi Saito, Hiroaki Kumano, Mohammad Ghazizadeh, Chigusa Shimokawa, Hideki Tanemura

**Affiliations:** 1 Comprehensive Research Organization, Waseda University, Tokyo, Japan; 2 Faculty of Human Sciences, Waseda University, Saitama, Japan; 3 Logos Science Corporation, Tokyo, Japan

**Keywords:** Acceptance and Commitment Therapy, psychogenic erectile dysfunction, smartphone app

## Abstract

**Background:**

Erectile dysfunction (ED) is a multifactorial disorder with both psychogenic and organic components, but psychosocial factors are usually neglected.

**Objective:**

The purpose of this study was to develop a smartphone application targeting psychosocial factors of ED and to examine its feasibility, acceptability, and treatment response to determine the parameters for a larger clinical trial.

**Methods:**

In this single-arm feasibility study, 8 participants with situational ED were enrolled. Dr. App, a newly developed smartphone treatment application for patients with psychogenic ED consisting of 8 weekly modules based on Acceptance and Commitment Therapy, was delivered. The primary outcome was comparison of the International Index of Erectile Function-15 domain scores measured pre- and post-intervention.

**Results:**

Six out of 8 participants completed the Dr. App and the post-intervention measures. The Wilcoxon signed-rank test showed a significant change in erectile function (*P* < 0.05; *r* = –0.65) and a significant trend in intercourse satisfaction (*P* < 0.10; *r* = –0.47) and overall satisfaction (*P* < .10; *r* = –0.47). Additionally, the reliable change index values were used to calculate the number of participants for whom a clinically meaningful difference occurred. The results showed that 33.30% of the participants had clinically meaningful differences in erectile function and 66.70% in intercourse satisfaction and overall satisfaction. On the other hand, no significant differences were shown in orgasmic function and sexual desire.

**Conclusions:**

Findings from this study support the feasibility, acceptability, and potential usefulness of the smartphone application targeting psychosocial factors of ED and warrant a larger randomized clinical trial to confirm the results.

## Introduction

Erectile dysfunction (ED) is a major health problem causing significant distress and negatively influencing men's quality of life and their partners.^1^ According to the biopsychosocial model, ED is often multifactorial, combining psychogenic and organic components.^2^ Nonetheless, the fact is that medical treatments only manage the organic factors influencing erectile function and neglect the psychological factors.^3^ Pharmacological treatment doesn't respond to all the co-occurring aspects of ED, including anxiety, loss of self-confidence, depressed mood, difficulties in a couple's communication, relationship disputes, or a partner's sexual problems.^4^ This is particularly true when the marital and intrapsychic components are primarily involved in the pathogenesis of ED.

Internet-based therapy can provide psychological treatment to men seeking ED treatment but are too afraid or shy to solicit support in person. A few studies suggest that psychological treatment for ED can be feasible and efficacious when delivered via internet-based cognitive behavioral therapy.^5^^,^^6^ In recent years, many smartphone apps developed for depression and anxiety have shown efficacy comparable to in-person treatment,^7^ but no apps targeting psychogenic ED have been available. This study aimed to develop a smartphone application targeting ED's psychosocial factors and examine its acceptability, feasibility, and treatment response to determine the parameters for a larger clinical trial. The hypothesis was that the smartphone application would be feasible and acceptable among this tested group.

## Materials and Methods

### Participants

This study was approved by the Waseda University Academic Research Ethical Review Committee. The study was also registered at the Japanese Clinical Trial registration (No. UMIN000047183). A total of 8 participants were recruited from March 2022 to May 2022 at an outpatient ED clinic in Tokyo, Japan.

The recruitment of patients was through a banner posted at the clinic's website. The patients with ED interested in participating in the study visited the clinic and completed the registration to participate. No financial incentives were paid for participation in the study.

Eligibility criteria for participation consisted of the following: those aged 20 to 59 years and those who meet the diagnostic criteria for situational ED (limited to specific types of stimulation, situations, or partners) according to Diagnostic and Statistical Manual of Mental Disorders-5 (ie, the experience of at least 1 of the following 3 symptoms in almost all or all [75%–100%] sexual activity: marked difficulty in obtaining an erection during sexual activity, marked difficulty in maintaining an erection until the completion of sexual activity, or marked decrease in erectile rigidity).^8^

Also, the above symptoms have persisted for approximately 6 months and have caused significant distress to the individuals. Those with mild to moderate ED (12–21 points) on the International Index of Erectile Function 5 (IIEF-5) score^9^ and are capable of morning erection and situational erection. Besides, the exclusive criteria were as follows: those receiving or attending any other psychological treatment or clinical trials; those with a diagnosis of hypertensive disease, heart disease, cerebrovascular disease, chronic kidney disease, or diabetes mellitus; those with suspicions of alcohol or drug abuse; those whose participation judged inappropriate for any other reason by their primary care physician. All participants complained of ED lasting for at least 6 months. They had no sexual dysfunctions other than situational ED. Informed consent was obtained from each participant before participation in the study.

### App description

We developed a new smartphone application targeting psychosocial factors of ED named Dr. App (Logos Science Corp, Tokyo, Japan). It consists of 8 weekly modules delivered via the Internet. All programs were developed based on Acceptance and Commitment Therapy (ACT).^10^^,^^11^ The efficacy of ACT has been evaluated in many randomized controlled trials investigating a broad range of target conditions.^12^ The programs were delivered in a variety of ways, including video, audio, chat-bot, freetext, and quiz formats. These could be completed within 10 to 15 minutes per day. Participants were provided an average of 12 programs. Participant and therapist had no face-to-face or telephone contact during the treatment. Module 1 provided education on the biopsychosocial factors of ED. In Module 2, education on the psychological flexibility of ACT was provided. Afterward, by following the stories of fictious ED participants, the participants were able to learn the relationship between psychological flexibility and ED. During Modules 1 and 2, participants were also asked to answer various questionnaires to assess psychosocial factors of ED (eg, depression, anxiety about sexual performance, relationships with sexual partners, and lifestyle). In Module 3, exercises on acceptance, mindfulness, and value clarification were conducted to increase psychological flexibility. From Module 4 onward, the most effective program for the participant was automatically selected based on previous assessments. For example, participants with high anxiety about sexual performance were offered attention training technique.^13^ On the other hand, participants experiencing poor communication with their partners were offered; for example, sexual relationship building.^14^ From Module 6 onward, exposure tasks designed to expose the patient to sexual interaction with their partner were provided.^15^

## Measures

### Primary outcome

The Japanese version of the IIEF-15 is a 15-item self-report questionnaire designed to measure ED and several other dimensions of male sexual functioning, which consist of 5 domains^9^: erectile function (EF), intercourse satisfaction (IS), orgasmic function (OF), sexual desire (SD), and overall satisfaction (OS). The primary outcome of this study was a change in the IIEF-15 domain scores from pre- to post-intervention.

### Statistical Analysis

This study preferred nonparametric tests due to few samples and the inability to meet normality assumptions on some IIEF-15 domains. The Wilcoxon signed-rank test was used to determine the difference between mean values of rank obtained from pre- and posttest measurements. For each IIEF-15 domain, *P* < 0.05 was considered statistically significant, and *P* < 0.10 was considered a tendency. The effect size was calculated, small, medium, and large effect power *r* < 0.20, 20 < *r* < 50, and *r* > 50, respectively.^16^

Additionally, the reliable change index (RCI) was utilized to determine if the changes induced by Dr. App were of clinical significance.^17^ We calculated the proportion of participants with an RCI ≥1.96. Data were analyzed using IBM SPSS Statistics version 25 (IBM-SPSS Inc, Armonk, NY) and HAD18_00218.^18^

## Results

### Participant characteristics

A total of 8 participants (mean [SD] age = 42.83 [13.19] years) were included (Table 1). The participants generally were highly educated, had busy work schedules, and tended to sleep less. All participants were married or had been living with a heterosexual specific partner. Additionally, they were not taking phosphodiesterase-5 inhibitors.

**Table 1:** Demographic characteristics of participants (N = 8).

Characteristic	Result
Age, y*	42.83 (13.19)
Marital status†	
Married	7 (87.50)
Not married	1 (12.50)
Education† [n, (%)]	
High school or higher [n, (%)]	8 (100)
Employment status	
Employed	7 (87.50)
Unemployed	1 (12.50)
Working†	
<7 h, 7–9 h	1 (14.28)
>9 h	6 (85.72)
Smoking†	
Smoker	1 (12.50)
Former smoker, nonsmoker	7 (87.50)
Drinking†	
≥7/wk	2 (25.00)
Nondrinker to <6/wk	6 (75.00)
Sleeping†	
7–9 h	1 (12.50)
<7 h, >9 h	7 (87.50)
Exercise†	
≥1/wk	3 (12.50)
Do not exercise, sometime	5 (87.50)

⁎ Values are presented as mean (SD).

† Values are presented as n (%).

**Figure 1:**
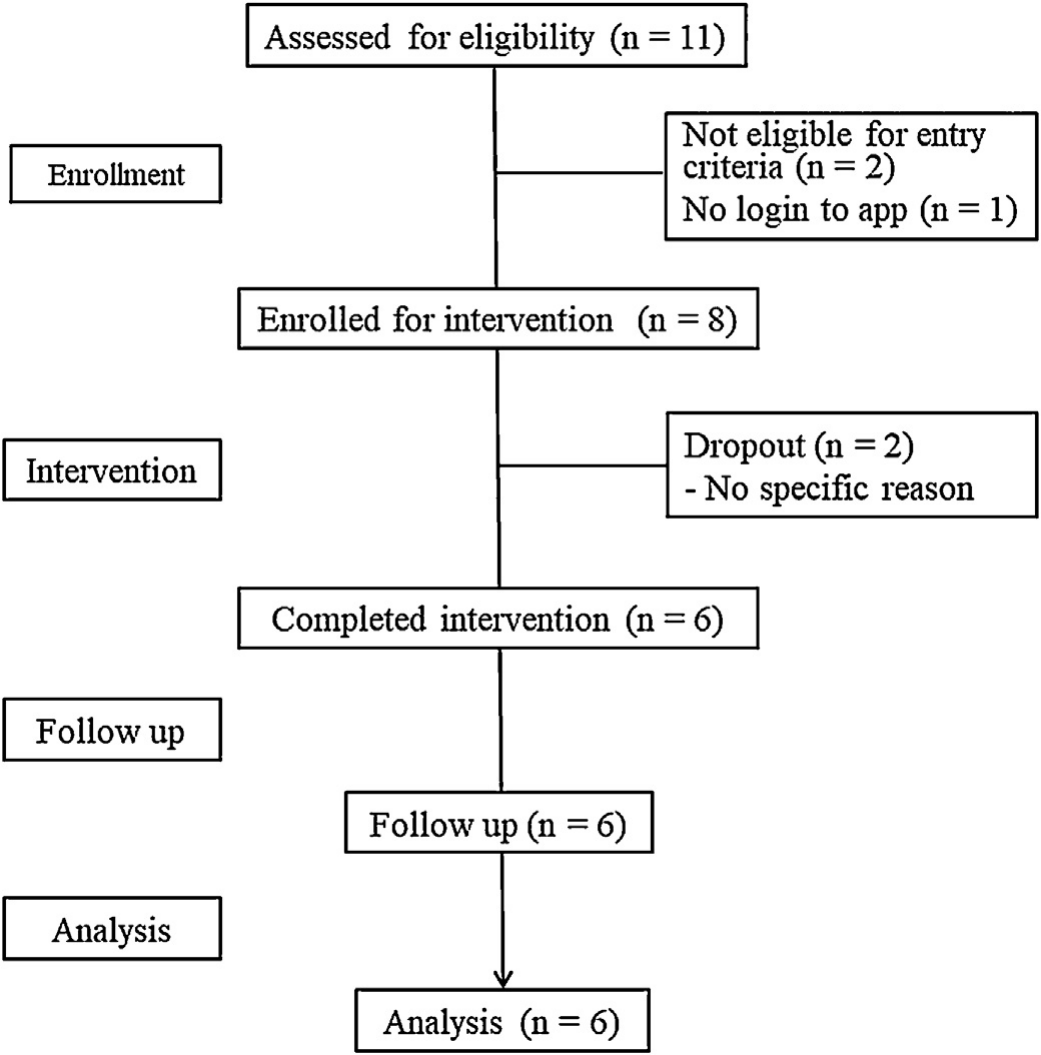
Consolidated Standards of Reporting Trials flow chart of the clinical trial.

Six participants completed the Dr. App and the post-intervention measures (Figure 1). Two participants dropped out during Modules 1 and 2. Five of the 6 completers responded to the questionnaire about using Dr. App. Some examples of responses included: “The app was reassuring because there are people other than doctors who understand me,” “My relationship with my partner has deepened,” “I had a strong impression of the study so that I would have preferred something more frank,” and “I would like to see a pop-up notification every day or something to keep me going.”

### Primary outcomes

**Figure 2:**
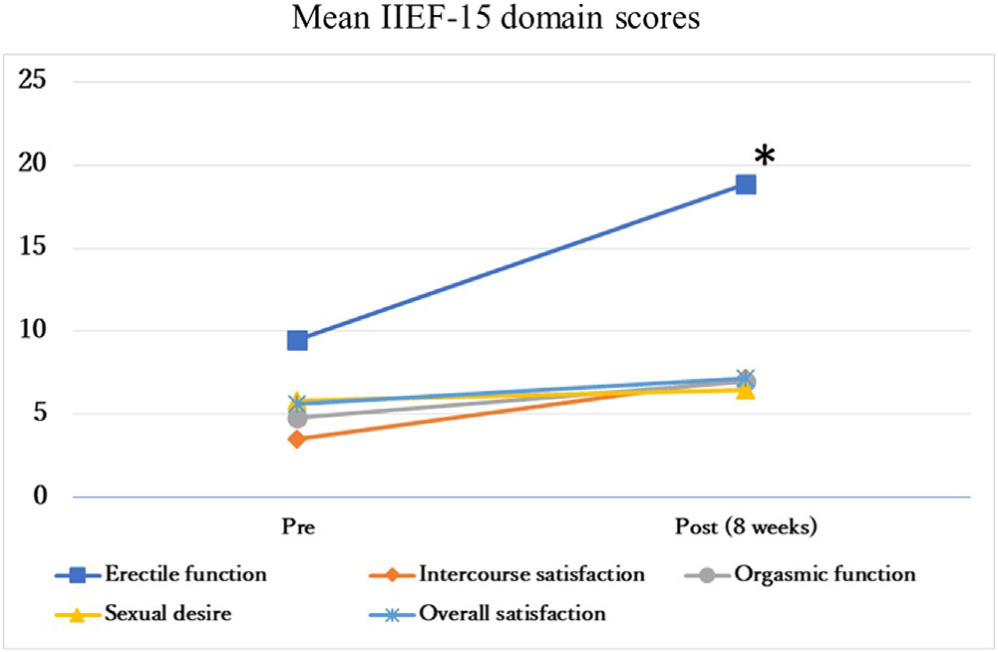
Changes in the mean of each International Index of Erectile Function-15 (IIEF-15) domain scores for all participants pre- and post-intervention. The Wilcoxon signed-rank test showed a significant change in the erectile function (*P* < .05; *r* = –0.65) and a significant trend in intercourse satisfaction (*P* < .10; *r* = –0.47) and overall satisfaction (*P* < 0.10; *r* = –0.47). *The Wilcoxon signed-rank test (*P* < 0.05).

Changes in IIEF-15 domain scores for each participant are shown in Table 2 and Figure 2. The Wilcoxon signed-rank test showed a significant change in the EF (*P* < 0.05; *r* = –0.65) and a significant trend in the IS (*P* < 0.10; *r* = –0.47) and OS (*P* < 0.10; *r* = –0.47). Additionally, RCI values were used to calculate the number of participants for whom a clinically meaningful difference occurred. The results showed that 33.30% of the participants had clinically meaningful differences in EF and 66.70% in IS and OS. On the other hand, no significant differences were shown in OF and SD.

**Table 2:** Changes in International Index of Erectile Function-15 domains scores for each participant.

	Pre (n = 6)	Post (n = 6)	*P* value	Effect size (*r*)
Erectile function				
Mean (SD)	9.50 (7.94)	18.83 (9.70)	0.03	–0.60
Patient 1	3	20		
Patient 4	19	25		
Patient 5	10	27		
Patient 6	19	27		
Patient 7	1	4		
Patient 9	5	10		
Intercourse satisfaction				
Mean (SD)	3.50 (4.46)	7.16 (4.16)	0.10	–0.47
Patient 1	0	8		
Patient 4	9	12		
Patient 5	3	9		
Patient 6	9	9		
Patient 7	0	0		
Patient 9	0	5		
Orgasmic function				
Mean (SD)	4.83 (5.30)	7.00 (3.79)	0.27	–0.31
Patient 1	0	7		
Patient 4	9	10		
Patient 5	10	10		
Patient 6	10	9		
Patient 7	0	0		
Patient 9	0	6		
Sexual desire				
Mean (SD)	5.83 (3.12)	6.50 (3.20)	0.58	–0.15
Patient 1	4	6		
Patient 4	9	10		
Patient 5	6	10		
Patient 6	10	7		
Patient 7	2	2		
Patient 9	4	4		
Overall satisfaction				
Mean (SD)	5.66 (1.96)	7.16 (1.60)	0.10	–0.47
Patient 1	6	6		
Patient 4	8	10		
Patient 5	6	8		
Patient 6	6	7		
Patient 7	2	6		
Patient 9	6	6

## Discussion

Findings from this study support the potential usefulness of an ACT smartphone application targeting ED with respect to feasibility, acceptability, and treatment response. Six of the 8 participants (75%) completed the intervention, which is higher than the 8% to 50% completion rate observed for internet-based cognitive behavioral therapy for ED in previous studies.^5^^,^^6^ The usefulness of interactivity and personalization with the chat-bot is suggested to increase engagement with the app.^19^ Incorporating these elements into the app may have prevented dropouts.

A recent systematic review shows psychological treatments benefit men with psychogenic ED, improving erectile function and overall sexual satisfaction.^20^ Although none of these studies included studies using apps, the results of this study suggest that similar effects may be achieved with apps. However, this study found no effect on the IIEF-15 domain of OF and SD. It might have been challenging to improve only psychotherapy for OF and SD because of the greater involvement of biological factors in these domains.

It is generally assumed that ED in men younger than age 40 years is primarily driven by psychosocial factors, whereas ED in older men would be more biologically based.^21^ Of the 6 completers, 1 who showed little improvement in IIEF-15 score was in his late 50s and may not have been eligible for the content of this application.

The Dr. App smartphone intervention has the potential to overcome treatment barriers and increase availability of psychotherapy for patients with psychogenic ED. It may be used alone or in conjunction with standard pharmacotherapy to enhance the therapeutic effects. The strengths of this study are that it is a feasibility study assessing usability, acceptability, and treatment response of a newly developed smartphone application for alleviating psychogenic factors of ED. The limitations are the small sample size and single-arm design; thus, no conclusion about efficacy and generalizability can be made.

## Conclusions

This study evaluated the feasibility and acceptability of a smartphone application targeting psychogenic erectile dysfunction based on acceptance and commitment therapy (ACT). The results showed a positive effect of this approach to alleviating the psychosocial factors of ED. A larger randomized controlled trial considering a clinically significant effect in each IIEF-15 domain between pre- and post-intervention is currently being planned to follow the success of this feasibility study and to rigorously evaluate the effectiveness of this innovative smartphone app therapy.

## Declaration of Competing Interest

This study was funded by Logos Science Corp Ltd, Tokyo, Japan.

M. Ghazizadeh, C. Shimokawa, and H. Tanemura are members of Logos Science Corp, Ltd. The authors have indicated that they have no other conflicts of interest regarding the content of this article.
